# Neglected Anterior Shoulder Dislocation Treated With Open Reduction and Latarjet Procedure: A Case Report

**DOI:** 10.7759/cureus.35347

**Published:** 2023-02-23

**Authors:** Saber Zari, Otmane Sammouni, Najib Abdeljaouad, Hicham Yacoubi

**Affiliations:** 1 Traumatology and Orthopedics, Mohammed VI University Hospital, Oujda, MAR

**Keywords:** open reduction, latarjet procedure, neglected, shoulder, anterior dislocation

## Abstract

According to the literature, cases of neglected anterior shoulder dislocation are extremely rare, which still presents diagnostic and treatment challenges. An extensive surgical procedure is required for their treatment. This situation is still challenging, and there is currently no accepted therapeutic protocol to resolve it. We report the case of a 30-year-old patient who had a right shoulder trauma with an unnoticed antero-medial dislocation. The treatment established was an open reduction combined with the Latarjet procedure followed by good results.

## Introduction

A neglected shoulder dislocation (NSD) is a rare entity in orthopedics, with only a few cases being reported in the literature. Neglected anterior shoulder dislocations (NASD) are less common than neglected posterior shoulder dislocations (NPSD) by about one-third for two-thirds, respectively, however, in some literature, their numbers are sometimes higher than NPSD [1,2]. In some cases, these conditions go unnoticed or present with a proximal humerus fracture, especially when treated by unqualified professionals who often work in rural areas. Although most of these chronic cases are treated with open reduction, there is no established protocol for NASD due to a lack of research [3]. There are very few published cases of shoulder dislocations that have been neglected and have retained the full range of motion (ROM).

## Case presentation

**Figure 1:**
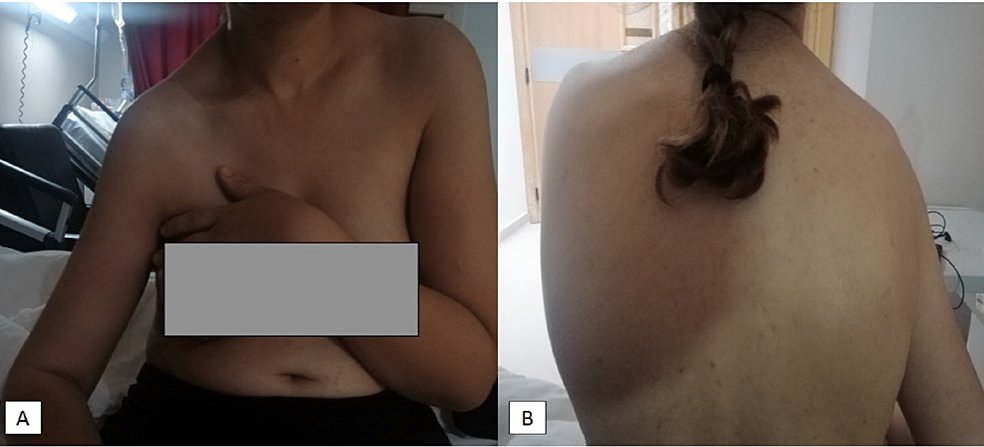
Clinical images showing (A) drooping right shoulder with a collapse of the shoulder stump, and (B) atrophy of the supraspinatus and infraspinatus fossa of right shoulder

**Figure 2:**
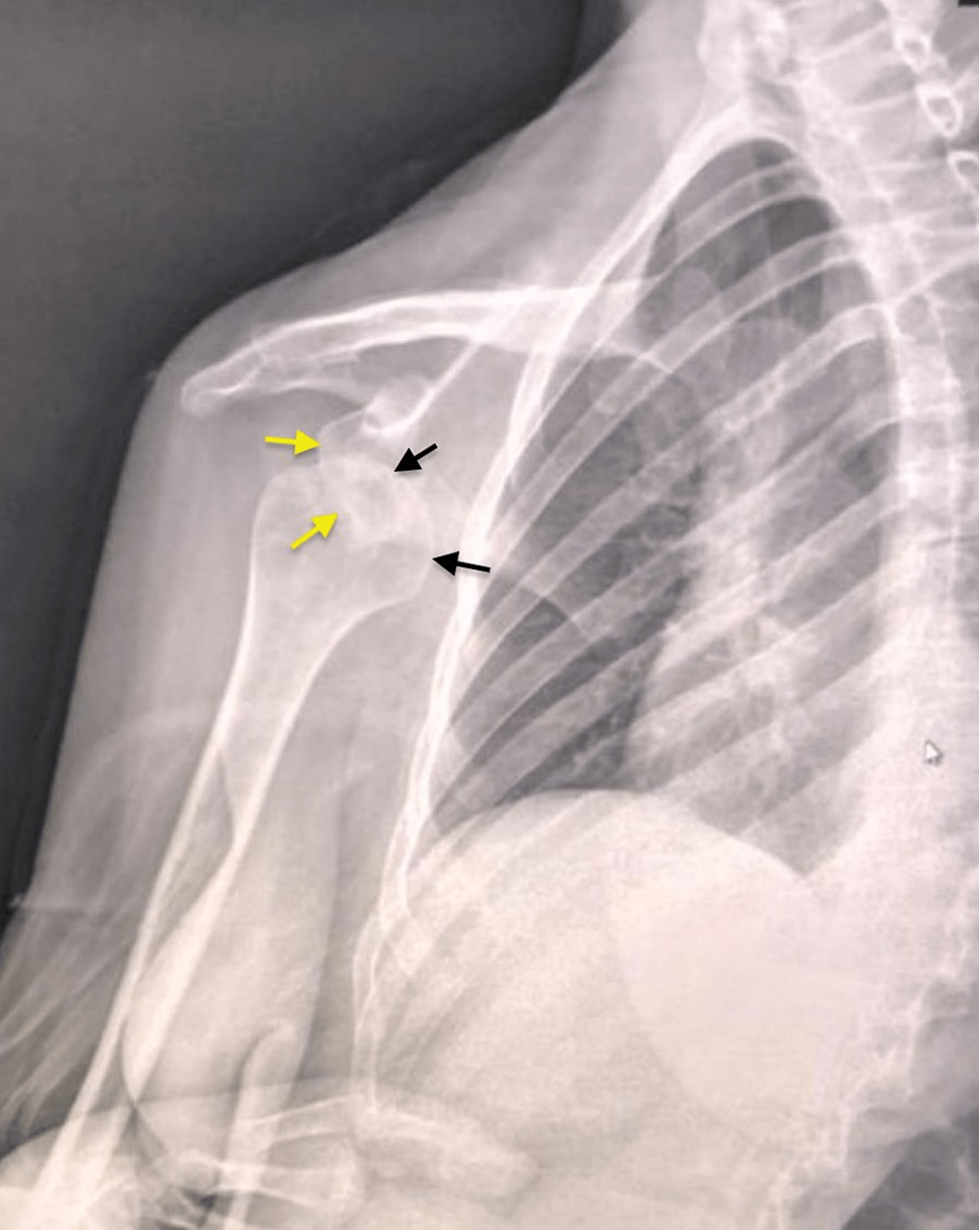
Frontal preoperative X-ray showing anterior dislocation of the right shoulder since three months in a 30-year-old patient. Also seen is the contact loss between the humeral head (black arrows) and shoulder blade glenoid cavity (yellow arrows).

Here we report the case of a 30-year-old right-handed patient admitted to our department for pain and functional disability of the right shoulder three months after a domestic fall. The patient initially consulted an unqualified practitioner who immobilized her shoulder after many manipulations. The evolution was marked by the progressive disappearance of pain with persistent ROM limitation. Physical examination found a right shoulder deformity with shoulder stump collapse and muscle atrophy (Figure 1), without neurologic signs of brachial plexus compression. The ROM of the right shoulder was: extension-flexion 25° to 70°; external-internal rotation 35° to 20°; and adduction-abduction was 30°to 40°. An anteroposterior (AP) radiograph of the right shoulder showed an anteromedial dislocation of the right humeral head (Figure 2). Considering a neglected shoulder dislocation in a young patient, open reduction and bone blocking using the Latarjet procedure were indicated. 

**Figure 3:**
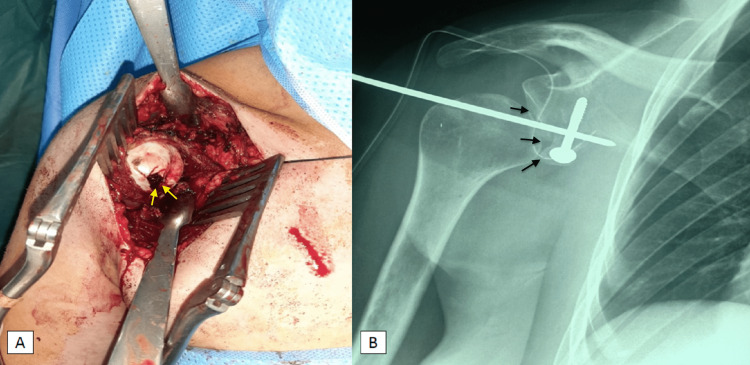
(A) Intraoperative view showing the bone defect on the right humeral head (yellow arrows) with loss of contact between the humeral head and the glenoid cavity. (B) Frontal postoperative X-ray showing reduced glenohumeral joint (black arrows) fixed with a Kirschner wire and one screw in accordance with the Latarjet procedure.

The operation was performed in the beach-chair position. After preparation and routine draping, a deltopectoral approach was made along the anterior axillary line followed by the identification of the deltopectoral groove and the cephalic vein, transection of the coracoacromial ligament 1.5 cm from its insertion in the coracoid, and release of the pectoralis minor off the coracoid with individualization of the conjoint tendon, osteotomy of the coracoid process at the vertical and horizontal junction, splitting of the subscapularis at the junction of the upper 2/3 and lower 1/3, and the realization of a capsulotomy. On exploration, the anteromedial dislocation of the humeral head was detected with wearing of the humeral head. After removing fibrotic tissue, a reduction of the dislocation and placement of an arthrosis Kirschner wire was made followed by the Latarjet procedure by placing the graft and fixing it to the anteroinferior border of the glenoid with a 4.5mm malleolar screw (Figure 3 A) [4]. Postoperatively, the glenohumeral joint was reduced. The postoperative radiograph showed a good position of the bone block with a reduced shoulder joint (Figure 3 B). We removed the K-wire three weeks after surgery, and the patient began an appropriate rehabilitation program.

**Figure 4:**
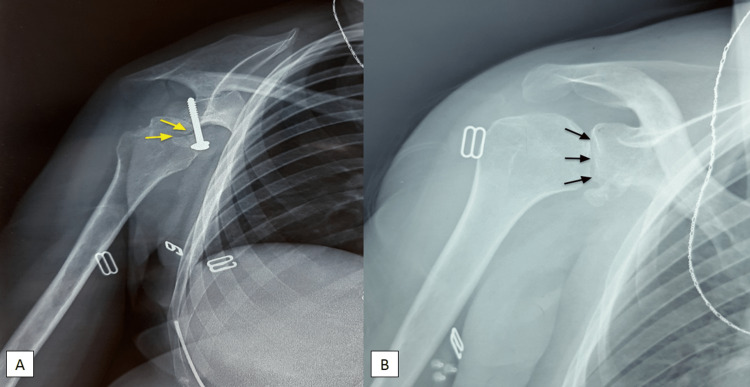
(A) Frontal X-ray showing pseudarthrosis of the coracoid bone block after four months of the Latarjet procedure (yellow arrows) with subluxation of the humeral head. (B) Frontal X-ray one month after removal of the screw showing a glenohumeral joint in place (black arrows).

**Figure 5:**
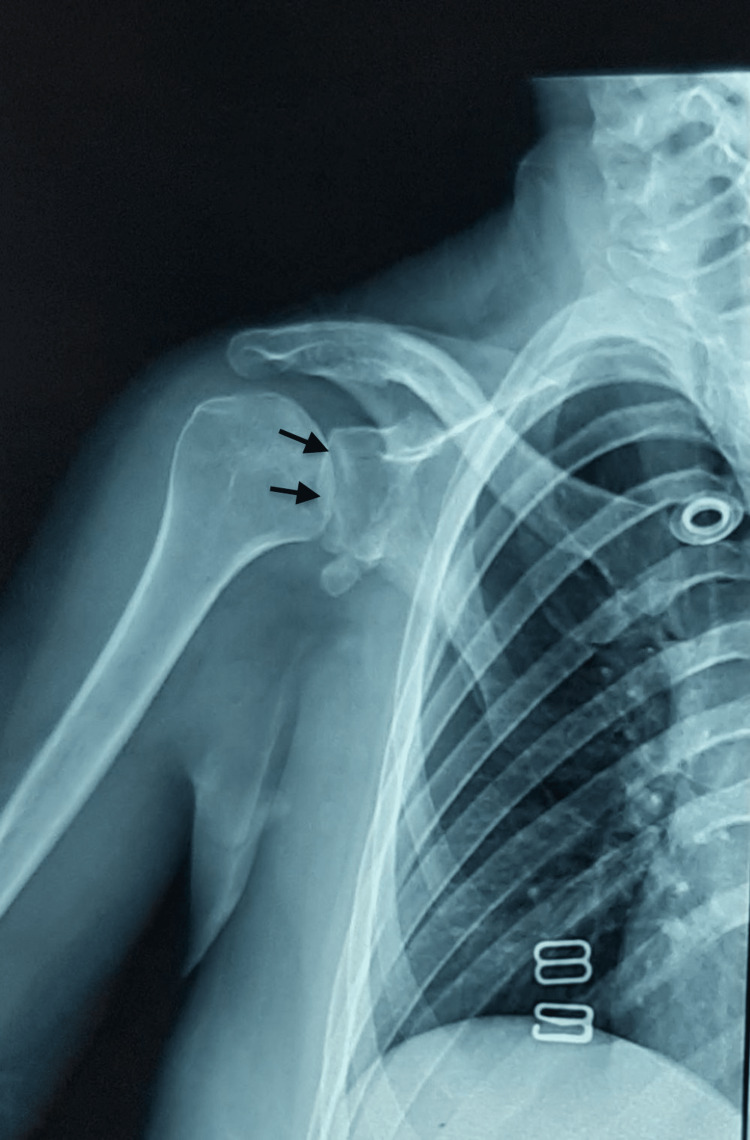
Frontal X-ray one year after the Latarjet procedure showing a good position of the glenohumeral joint (black arrows)

**Figure 6:**
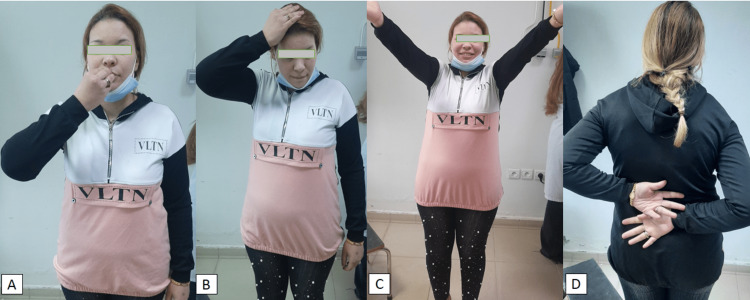
Functional results at the last follow-up (A) Hand-to-mouth test, (B) Hand-to-head test, (C) Patient with flexion of the right shoulder similar to the contralateral side, (D) Patient with internal rotation of the right shoulder similar to the contralateral side

Four months after surgery, the patient presented a pseudarthrosis of the coracoid bone block. We removed the screw with an adapted rehabilitation protocol (Figure 4). The latest follow-up shows a good position of the graft, good congruence of the glenohumeral joint (Figure 5), and articular mobility was preserved with no recurrent dislocation (Figure 6).

## Discussion

Neglected anterior shoulder dislocations are rare. However, in some series in the literature, their frequency is not negligible. In the series by Rowe (&) Zarins, anterior dislocations represent 65% of chronic dislocations compared to 35% of posterior dislocations [2]. The term "neglected shoulder dislocation" is used to describe a situation in which the injury goes unrecognized for at least three weeks or four weeks [2].

The etiologies are in most cases traumatic, but sometimes post-convulsive. It may also be the consequence of unmonitored immobilization after reduction of an anterior dislocation, after immobilization with too much abduction, or muscular sideration with post-traumatic hemarthrosis, all allowing the humeral head to refluxate in an anteroinferior position. An unsuspected glenoid fracture, affecting more than the anterior third of the articular surface, may cause a recurrence, which may be missed in the absence of control [5].

An NASD is a challenging condition for patients and professionals to manage due to bone deficiencies and significant soft tissue damage such as Hill-Sachs and Bankart lesions, massive glenoid bone loss, rotator cuff tears, and later severe glenohumeral osteoarthritis which may also be present [6,7]. Proposed techniques such as Bankart repair, coracoid transfer, bone grafting, and arthroplasty are varied and difficult to analyze because series are extremely limited. Reduction by the deltopectoral approach followed by stabilization is the most adopted procedure in the most recent series [5]. Perniceni reported three cases of open reduction followed by a costal graft with good results [8]. Mansat reported five cases of open reduction followed by Bankart surgery with one excellent, three good, and one poor result [9]. Goga reported 10 cases of Latarjet bone block protected by acromiohumeral K-wire, with eight excellent or good results in 10 cases [1]. In the study by Deepak et al., who performed open reduction with Latarjet procedure in a patient with six months of glenohumeral dislocation, the results at the last follow-up were encouraging, with no new episodes of dislocation [10]. In our case, we performed the Latarjet procedure to prevent recurrent anterior instability.

Recurrent instability may result from the bone defect, depending on the size and depth of the defect. In the presence of such instability, rotation osteotomy of the proximal humerus, partial or complete humeral head replacement is recommended for defects that represent more than 40% to 50% of the head [10]. The Latarjet procedure and non-anatomic procedures such as bone or soft tissue transfer (remplissage) of the infraspinatus are recommended for defects greater than 25% but less than 40%; these techniques include allograft repair of the head and humeral head disimpaction/humeroplasty [11]. The Latarjet procedure is more comfortable for the surgeon than the remplissage operation and offers stability through its "triple effect." The Latarjet technique for the treatment of NASD is validated for its ability to treat recurrent anterior shoulder dislocations with or without significant glenoid bone defects [12].

## Conclusions

Neglected anterior dislocations of the shoulder are serious and rare injuries that are usually poorly tolerated and difficult to manage. Different therapeutic attitudes have been proposed, from open reduction to surgical abstention. However, open reduction and the Latarjet technique have been shown to have an excellent success rate and prevention of recurrence.
